# On the Deposition Process of Ceramic Layer Thin Films for Low-Carbon Steel Pipe Protection

**DOI:** 10.3390/ma15134673

**Published:** 2022-07-03

**Authors:** Stefan Irimiciuc, Marius Gabriel Zaharia, Ramona Cimpoesu, Georgiana Bulai, Silviu Octavian Gurlui, Nicanor Cimpoesu

**Affiliations:** 1 Laser Department, National Institute for Lasers, Plasma and Radiation Physics, 077125 Bucharest, Romania; stefan.irimiciuc@yahoo.com; 2 Faculty of Material Science and Engineering, Gheorghe Asachi Technical University of Iasi, 59A Mangeron Bld., 700050 Iasi, Romania; marius_marius_zaharia@yahoo.com (M.G.Z.); nicanor.cimpoesu@tuiasi.ro (N.C.); 3 Spectroscopy and Laser Laboratory (LOASL), Faculty of Physics, Atmosphere Optics, Alexandru Ioan Cuza University of Iasi, 11 Carol I Bld., 700506 Iasi, Romania; dascalu.georgiana@yahoo.com

**Keywords:** ceramic thin film, pulsed laser deposition, optical emission spectroscopy, electron temperature, ICCD imaging, plasma structuring

## Abstract

Ceramic thin films with variable thicknesses have been used in many applications. In order to protect the petroleum transportation pipes against the harmful H_2_S action, two ceramic materials as thin layers are proposed. In this article, pulsed laser deposition (PLD) of ceramic layers by in situ time-resolved optical techniques is investigated. Two ceramic materials were used as targets and real-time monitoring of the PLD process was realized via ICCD fast camera imaging and optical emission spectroscopy. The space–time displacement of the ceramic emissions was analyzed in order to determine the plasma structure and respective kinetic energies. Spectral-resolved investigation allowed the determination of plasma species individual velocities (in the first case: 43 km/s for C ionic species, 11 km/s for Si, from 25 to 5 km/s for atomic species; in the second case: 32 km/s for C ionic species, 11 km/s for W species, and 15 and 53 km/s for neutral species). SEM and AFM techniques were implemented to analyze the resulting ceramic layers showing homogeneous surfaces with characteristic material droplets. The ablation crater also reveals selective ablation during the deposition process. EDX results show that Al/Si is retained in the thin films similar to the target composition.

## 1. Introduction

Carbon steel and low-alloy steel are the most used materials for manufacturing equipment in the gas and oil industries, including tanks, radiators, heat exchangers, filters, ball valves, pipelines, and flanges, for satisfactory mechanical properties and a low cost. In the petroleum and gas industries, steels are used for different applications and are constantly exposed to different environments. The factors that influence the resistance corrosion of the material are: the concentration of hydrogen sulfide (H_2_S) or the dissolved concentration in the aqueous phase (pH), the presence of oxygen, sulfur or other oxidants, high pressure, high temperature, exposure time, as well as complex mechanical conditions [1,2]. The effect of hydrogen sulfide (H_2_S) on natural gas pipelines is little known in the short term and only presumed in the long term (years). Corrosion analysis of pipes used for the transport of natural gas containing a medium or high percentages of H_2_S was carried out and the poor properties of metallic materials used in the gas industry were shown [3]. H_2_S corrosion of some alloys can be anticipated, but due to the high toxicity of this compound, the available experimental results are limited. Corrosion studies of the effect of H_2_S on some carbon steels at a laboratory scale and with safety facilities have been reported in the literature [3,4]. Another important aspect in petroleum and gas transportation pipes is the hydrogen-induced fracture surface of a steel lath [5,6] that can be diminished or removed by applying protective ceramic layers.

Coating technology has become a very effective way to improve the properties of carbon steel in recent years. The pulsed laser deposition (PLD) technique is suitable for obtaining thin, compact and good stoichiometry layers that can protect metallic materials from the harmful action of H_2_S [7].

The PLD process can be used to deposit thin films with good uniformity of composition and film thicknesses on large substrates. Recent developments in the PLD process expand the deposition area by up to 4 inch, which is suitable for industrial applications [8]. New products and equipment are now being marketed at a steady pace for both research and production. There are applications that require high efficiency for standard wafers and long lengths of covered conductors [9].

Even though the use of PLD has increased in the recent years as a major deposition technique, when it comes to complex stoichiometric thin films, controlling and tailoring conditions in order to achieve optimum film properties can be a difficult task. Therefore, the results can span from the generation of high-quality stoichiometric films as reported in [10] to non-stoichiometric films during PLD [11]. In recent years, a concerted effort has been put into implementing various diagnostic techniques including Langmuir Probe [12,13], optical emission spectroscopy [14,15], ICCD imaging and mass spectrometry [16] to control and understand the deposition process. The importance of monitoring the deposition process was shown in a series of papers, where the group of Geoghegan reported on complex structure formation for deposition at high background pressures [17,18,19]. The group of Lippert [20,21,22] reported on the kinetic energy distribution and oxidation process during the deposition of complex oxide films and the group of Schou [23,24,25,26,27] reported on the congruent transfer of target stoichiometry, scattering processes and particle distribution during the deposition process. New techniques based on additive manufacturing can be used to improve the corrosion resistance of transportation pipes [28,29,30] based on advantages including ease of use, the simple-to-meet requirements for its machinery and the possibility of manufacturing individual specimens cost effectively.

In this paper, we report on the deposition of ceramic protective coatings on metallic samples. Time-resolved optical emission spectroscopy was performed in global and spectral-resolved approaches. The structure, kinetics and evolution of each deposition process were investigated. The resulting films were analyzed by a surface investigation technique to verify the congruent transfer of the ceramic composition while the post-deposition investigation of the ablated crater was carried out to gather insight into the ejection of the multiple species present in the plasmas.

## 2. Methodology

Targets are made of ceramic materials similar to industrial materials used for high-temperature or metallic materials processing. The first sample was obtained from a mixture of Al_2_O_3_ and SiO_2_ powders and pressed into cylindrical pellets (at a ratio of approximately 50/50 wt%). The target samples were subjected to sintering treatment at 900 °C for 5 h. The final chemical composition of target 1 (C1) was determined by a EDX detector (average values from five determinations): O: 58.35 (detector error: 7.39%), Si: 21.5 (detector error: 1.09%) and Al: 20.15 (detector error: 1.15%) wt%. For the Al_2_O_3_ + TiO_2_ + WC (chemical composition determined through EDX: Al: 48.73 (detector error: 1.51%), O: 38.78 (detector error: 14.97%), Ti: 6.86 (detector error: 0.22%), C: 4.66 (detector error: 0.21%), W: 0.8 (detector error: 0.06%) and Co: 0.14 (detector error: 0.03%) wt%) target, we used a ceramic material designed for metallic sample processing as substrate.

Ceramic thin films were deposited by the pulsed laser deposition (PLD) technique. A detailed representation of the experimental setup can be seen in [14]. A 10 ns Nd:YAG pulsed laser (Quantel Brilliant EaZy, Edinburgh, UK), (*λ* = 532 nm and repetition rate = 10 Hz) was focused by a 40 cm focal point lens on two ceramic targets (C1—a Al_2_O_3_ + SiO_2_ mixture; C2—a Al_2_O_3_ + TiO_2_ + CW mixture). The spot diameter at the impact point was approximately 700 µm, while the energy of the laser beam was monitored by a FieldMAX II power meter (Coherent, Santa Clara, CA, USA) and set at 30 mJ/pulse, leading to a laser fluence of 3 J/cm^2^. The samples were placed in a vacuum chamber with a residual pressure of 6 Pa ensured by a 300 L/m dry scroll pump (Agilent TriScroll 300, Santa Clara, CA, USA). P265GH steel discs (10 mm diameter and 5 mm height) were used as substrates and were placed at 5 cm from the target. The deposition process was monitored using an ICCD camera: PI-MAX3 (SK Advanced Group, Kadima, Israel), 1024i with a variable gate time of 5 to 500 ns, placed orthogonally to the plasma expansion direction. Two scenarios were employed for in situ monitoring of the PLD process: ICCD fast camera imaging, centered around recording the overall emission of the plasma (each image containing information over events), and spectral, space- and time-resolved emission of the laser-produced plasma, which is based on recording each emission spectrum averaged over 500 events. For the optical emission spectroscopy measurements, a Princeton Instruments Acton 2750 system (Acton, MA, USA) with a spectral resolution of 0.02 nm was used. The time-resolved procedure involved a step-and-glue setting, which allowed the recording of the global emission spectra from a 600 μm-wide plasma volume (integration time 2 μs and a delay of 100 ns) centered on the main expansion direction in a 300–700 nm spectral range. The monitoring system was calibrated with two calibration sources (He-Ne and Hg). During the experiments, the target was continuously moved in a linear pattern to ensure fresh surface irradiation and to overcome possible crater formation and thermal damage to the target.

The ceramic targets and thin layer surface were analyzed using scanning electron microscopy/SEM Vega Tescan LMH II, Brno, Czech Republic, SE Detector, HV, Vega Tescan, Brno, Czech Republic) and atomic force microscopy (AFM, Easy scan II, NanoSurf, Liestal, Switzerland). Insights into the chemical composition of the targets and the ceramic layers were found using an EDX detector, Bruker, Xflash, Billerica, MA, USA. The automatic mode was used for elements identification and the mapping mode for elements distribution.

## 3. Real-Time Monitoring during PLD of Ceramic Protective Layer

**Figure 1:**
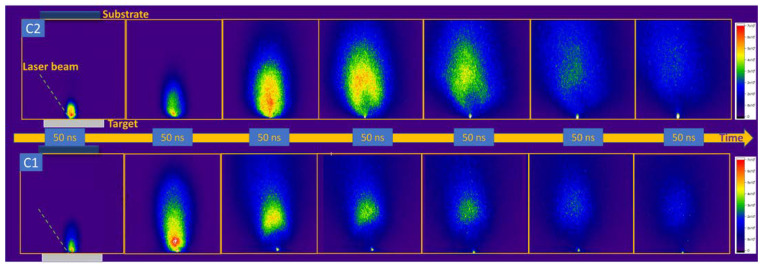
ICCD images of the laser-produced plasmas on C1 (Al_2_O_3_ + SiO_2_) and C2 (Al_2_O_3_ + TiO_2_ + WC) on a 1.5 μs time span.

The first step in monitoring the deposition process was to record a movie of the deposition process via ICCD fast camera imaging. This required recording the unresolved spectral light from the plasma and projecting the image of the plasma on the CCD detector of the camera. Snapshots from these movies are presented in Figure 1 for both investigated ceramics. The lifetime of the plasma (in terms of the emission) is approximately 1 μs for both ceramics. This results from a combination of the laser fluence value, background pressure and bonding energies within the ceramic structure. The three-parametric dependence will also be reflected in the expansion velocities of the plasma. As reported by our group in [31,32,33,34], the inner structure of the plasma can have a complex of two or more substructures which forms during expansion. Comparing the geometries of the two plasmas during the deposition process, we observ that the C1 plasma has a more elongated shape reaching the substrate (visible emission contribution to the understanding of the plasma volume) after 500 ns, whereas C2 takes approximately 1 μs having a more compact shape in the first stage of expansion. The long-term optical emission is more dominant for the C2 plasma when compared with C1, and this is due to the heterogeneity of the structures. C2 contains a wide variety of elements with different atomic masses (Al-27 amu, W-178 amu, Ti-, O-16 amu, C-8 amu, and Co-59 amu) while C1 has a simple structure containing only Al, Si, C and O. For the plasma containing heavier elements, as per our previous report from [35], there is a kinetic energy distribution favoring the expansion of lighter elements with a higher velocity. With such a complex stoichiometry in C2 plasma volumes, an increased number of collisions will occur, leading to an enhanced late time emission.

**Figure 2:**
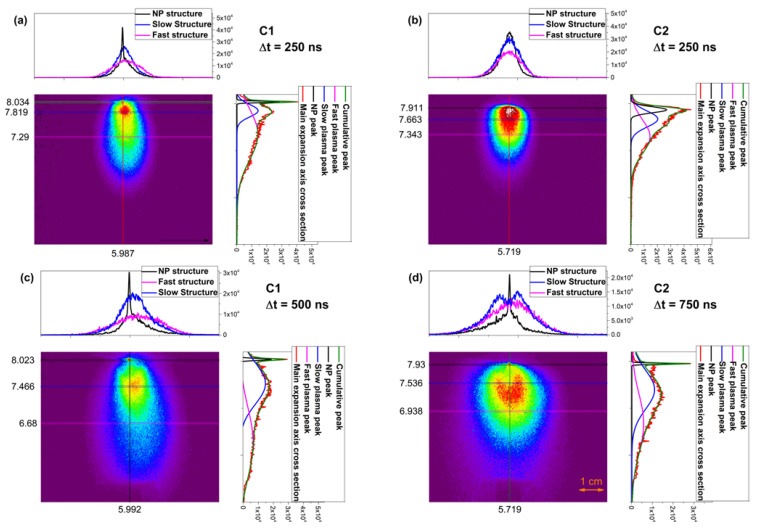
ICCD snapshots for C1 and C2 plasma at 250 (C1-(**a**) C2 (**b**)) and 750 (C2-(**d**) and 500 (C1-(**c**))ns delays with a cross section performed along and across several axis (NP peak—black curve, slow peak—blue curve, fast peak—magenta curve, and main expansion axis cross section—red curve).

In Figure 2, we have plotted representative pictures of the plasma with their respective cross section across several axes. We observe that the early-stage geometry of the plasma (Figure 2a,b) is, as expected, a feather-like shape, while the two plasmas differentiate considerably at later stages. C1 plasma has a more elongated shape, while C2 plasma has a larger emissive volume. A special feature is seen in the C2 plasma at the plasma core (in red, Figure 2d). The ICCD imaging also show that there are strong gradients along the main expansion axis but also across the same direction. It can also be seen that the core of the plasma has an arrow shape geometry, which is an indication of heterogenous distribution within the core containing species with different kinetic energies. In the axial direction (Figure 2a,b, red line), we observe for the C1 plasma a small peak in the proximity of the target, attributed to the presence of molecules and clusters in the plasma (NP), followed by a dominant peak (slow structure) and a secondary one with a reduced intensity (fast structure). The intensity ratio between the two structures is ½. For the C2 plasma, we observe a more confined structure, with clear contribution from all three structures. For late expansion times (Figure 2c,d), the identified structures are well defined and spatially stretched. On each structure, we performed a transversal cross section in order to investigate the angular distribution of each structure. The results are shown in the inset of Figure 2. All investigated plasmas present a structure with a narrow NP peak, while the fast structure has a wider distribution than the slower one due to the increased number of collisions and the density/energy gradients in the plasma. At longer evolution times for the C2 plasma, the slow structure is defined by a lateral plume splitting. These results, according to [25,36], can be related to the scattering effect and angular heterogeneity of the species within the investigated plasma volume. Since the C2 plasma contains several elements (Al, O, Ti, C, and W) with differences in the mass and size of the particles, a heterogeneous distribution is highly probable.

**Figure 3:**
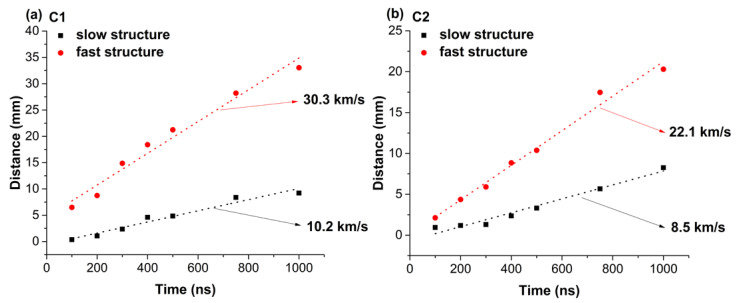
Space–time displacement of the two main emission maxima seen in ICCD fast camera imaging experiments. (C1-(**a**)); (C2-(**b**)).

By plotting the space–time displacement of the peaks extracted from Figure 2 and fitting this function, the expansion velocities (Figure 3) can be derived. The data define linear functions, which is an indication of the constant drift velocity of the plasma with negligible contribution from the residual atmosphere from the reaction chamber. The 3rd structure (slowest) is seen expanding at 2.41 km/s for C2 and 1.72 km/s for C1, and this means that the thermal effects are more damaging in the C2 sample as it ejects clusters with higher velocities. The 2nd structure (slow structure) is expanding at 8.5 km/s for C2 and 10 km/s for C1, while the 1st structure (fast structure) flows at 22.5 km/s for C2 and 30.3 km/s for C1. These differences are explained by the composition of the plasma as C1 has lighter elements which will gain more energy during the acceleration stages of expansion [37].

**Figure 4:**
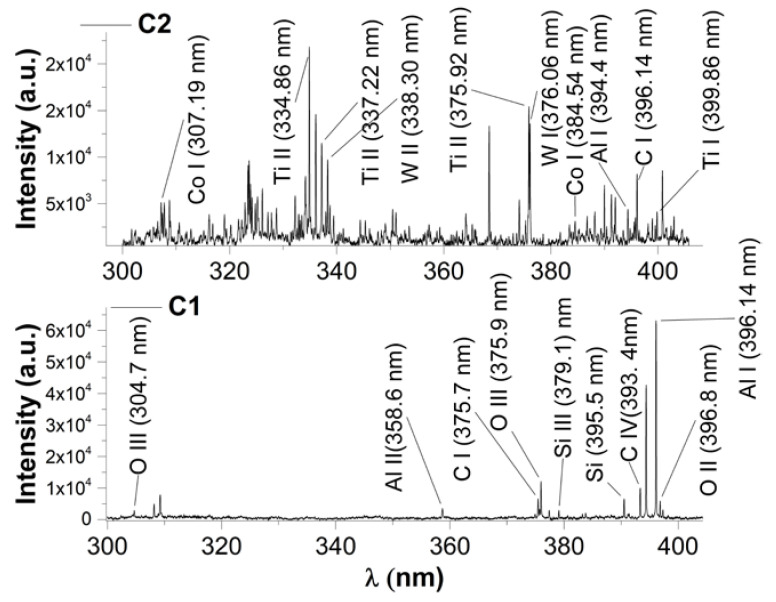
Optical emission spectra of the two investigated plasmas.

To obtain insight into the individual structure of the plasma, we performed optical emission spectroscopy with a long integration time along the full lifetime of the plasma. This choice is justified as some species (e.g., molecular ones) are seen only in specific time windows. In Figure 4, the optical emission spectra of the investigated plasmas is shown. The identification of the emission lines was performed using a specialized database [38], and it is noticeable that all the elements ejected from the target are seen in the emission. For C1 plasma, we observe higher ionized states for Si (Si III at 379.1 nm) and O (O III at 375.9 nm), which means that in the front of the plume lighter elements are found [39], with energies exceeding 18.8 eV, the secondary ionization potential of oxygen. In contrast, for C2 plasma, most ionizations are of 1st rank which means that the highest energies in the front are approximately 7–8 eV. The difference in the kinetic energies in the plasma is also seen from the ICCD fast camera imaging measurements. Using the Boltzmann plot technique from [40], we determined an average excitation temperature for each of the plasma, averaged on 100 ns, and determined as averages on the composing species. For C1 plasma, we found 0.5 eV, while 4.6 eV was determined for C2 plasma. These differences are in line with the ICCD fast camera imaging, where there is a considerable difference in the emissive volume of the two plasmas. The presence of a highly complex plasma structure with multiple composing species of C2 will also be reflected through an increase in the number of collisions and thus energy losses though excitation processes. The results are well correlated with the ICCD fast camera imaging determination of the plasma expansion velocity of each structure and confirms that although C2 plasma has lower kinetic energy, the balance is towards thermal energies in the plasma (collision, excitation, etc.). For the C1 plasma, the energy gain from the laser ablation process is mainly used for particle acceleration and ionization processes.

**Figure 5:**
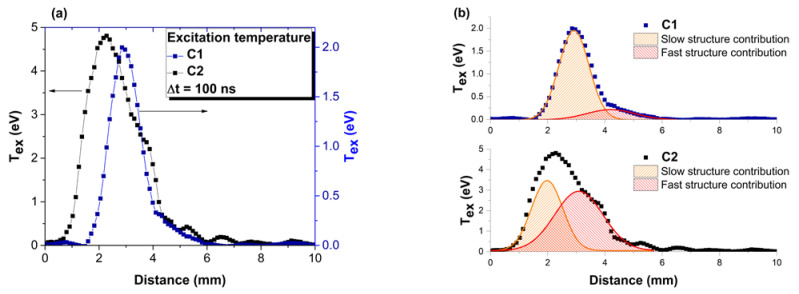
Spatial evolution of electron excitation temperature after 100 ns (**a**) and the deconvolution for both investigated plasmas (**b**).

To gain further insight into the plasma energy, sequential spectral-resolved images were recorded at various moments in time according to the procedure reported in [41]. In Figure 5, the spatial distribution of the electron excitation temperatures of the two investigated plasmas are shown. The excitation temperatures determined for the plasma generated in C2 are higher than those for the plasma generated in C1 by approximately a factor of 2. It can also be seen that the evolution depicts a first maxima at 2.1 mm from the target for C2 (4.6 eV) and at 3 mm for C1 (2 eV), followed by a second one at 3.7 mm for C2 (2.5 eV) and another at 4.5 for the plasma generated on C1 (0.3 eV). The multiple maxima evolution is in good agreement with the structure of the plasma seen through ICCD fast camera imaging and reinforces the idea of the plasma structuring into individual plasma substructures for which one can define local thermodynamic equilibrium conditions. The results also highlight the lack of any contribution from the NP structure towards the structuring of electron temperature in the time window chosen in this work. In [42], it is reported that the characteristic time of NP or heavier particles in laser-produced plasma is in the tens or hundreds of microseconds, in agreement with their lower expansion velocity. Our data show that the NP emission can be seen even in the early stages of ablation, and according to our global emission studies, there is no contribution to the electron excitation temperature spatial distribution.

**Figure 6:**
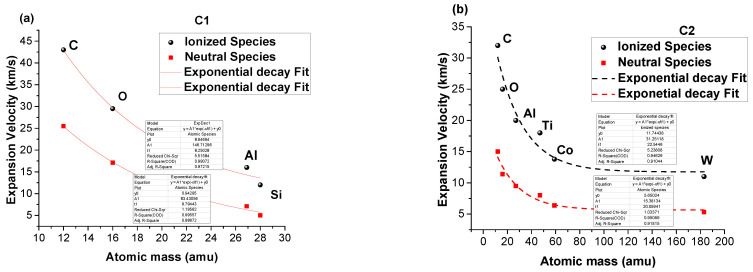
Individual expansion velocity evolution with the atomic mass for C1 (**a**) and C2 plasma (**b**).

Expanding on the approach presented above, we can determine the expansion velocity of each emission line by performing a linear fit of the space–time displacement of the maximum emission characteristic of each emission line. The resulting values are presented in Figure 6 as a function of the atomic mass of each species. For both investigated plasmas, the expansion velocity as a function of the atomic mass is defined by a decreasing trend, which can be approximated by a quasi-exponential decrease (shown in Figure 6b for C2 plasma). The calculated values for C1 plasma decrease from a maximum of 43 km/s for C ionic species down to 11 km/s for Si, and from 25 to 5 km/s for atomic species with a constant ratio between species; for the plasma generated on C2, 32 km/s for ionic C species, 11 km/s for W species, and 15 and 53 km/s for neutral species. Certain overall comparisons can be made between the two plasmas, especially on the common elements. The C and O species are more energetic in the C1 plasma when compared to the C2 plasma, which is due to the increased scattered process in the latter case, where heavier and larger species are present.

**Figure 7:**
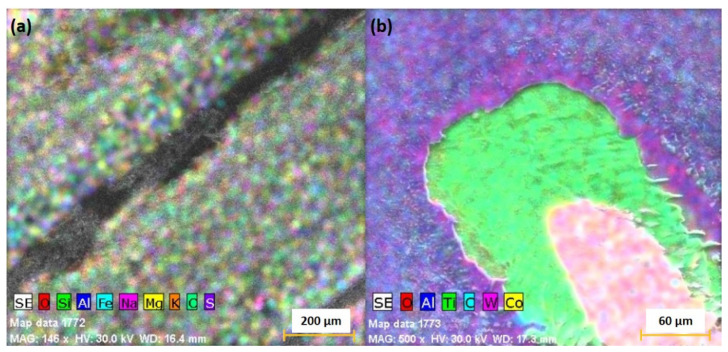
EDX mapping of the post-ablation crater of the C1 (**a**) and C2 (**b**) targets.

To assess the different scattering angles of the elements ablated from the two ceramics, we conducted an elemental analysis of the resulting crater. For the C1 target, there was a uniform distribution of the composing elements (with additional contamination of Na, K, S and Fe in small concentration Figure 7a). For the C2 target, a clear spatial distribution of the re-deposited material was observed. The results are represented in Figure 7b, where elements with a heavier mass such as W and Co are found in the center of the crater, which implies a small scattering angle, while elements such as Al, C or O are scattered towards the edge of the crater. This result is in good agreement with the reports from [24,38], where a similar distribution according to mass was seen for laser-produced multi-element plasmas.

The chemical composition of the ceramic layers (determined through EDX technique as an average value from five determinations of 1 cm^2^ areas) is strongly influenced by the substrate (the layers are thinner than 1 μm). For the layer obtained from the C1 target, the thin layer composition was: Fe: 84.8 (detector error: 1.23%), O: 6.25 (detector error: 1.16%), C: 4.98 (detector error: 0,87%), Al:1.54 (detector error: 0.12%), Si: 1.44 (detector error: 0.1%) and Mn: 0.96 (detector error: 0.1%) wt%. For C2, the thin layer composition was: Fe: 88.59 (detector error: 1.32%), C: 3.92 (detector error: 0.73%), O:3.81 (detector error: 2.42%), W: 2.37 (detector error: 0.11%), Co: 0.75 (detector error: 0.05%), Ti: 0.44 (detector error: 0.04%) and Al: 0.1 (detector error: 0.03%) wt%. Even though the influence of the substrate iron element is high, we observe a similar proportion of Al and Si to that obtained on the target.

**Figure 8:**
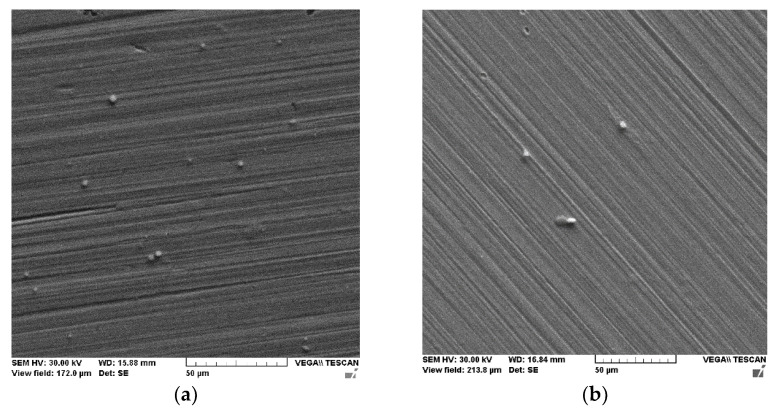

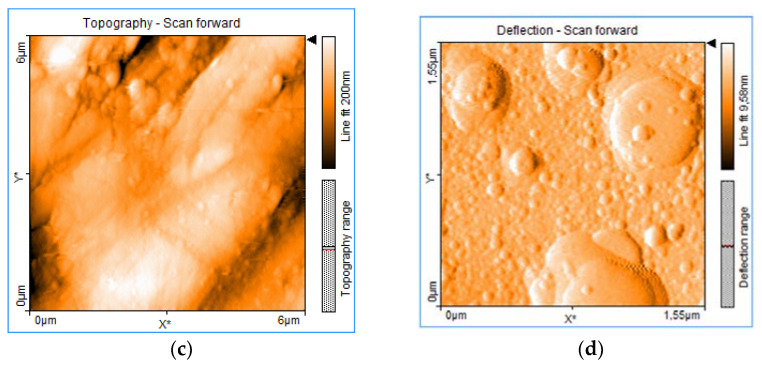
(**a**,**b**) SEM images of C1 and C2 layers and (**c**,**d**) AFM images of the ceramic layers obtained through PLD. (**c**) The surface layer obtained from the C1 target. (**d**) The surface layer obtained from the C2 target.

The quality of the ceramic thin layers (from targets C1 and C2) was observed through SEM and AFM, as shown in Figure 8a,b Figure 8c,d, respectively. The thin films are homogeneous and sporadically present few material droplets. The presence of droplets is in good agreement with the ICCD fast camera imaging in which a slower structure was seen. The μm droplets are not dominant and the films show a uniform distribution of the substrate. Quasi-stoichiometric transfer (some C impurities are seen for the C1) was achieved for both depositions, which shows that the structuring of the plasma is a local effect and does not affect the desired congruent transfer. The AFM images reveal different morphologies for the two films, with large molted pieces seen on the surface. Let us note that the rough nature of the coating does not affect the performance aimed for in these films.

The ablation process of the C2 target produces more droplets based on the chemical composition of the material (in addition to alumina, there are titania and tungsten carbide). In bulk, the C2 material presents a higher mechanical resistance and hardness due to the presence of carbide in the structure. The complex chemical nature of C2 contributes to the final structure of the film. The investigations on the deposition of multi-element protective coatings by PLD reveal complex plasma kinetics and structuring with influence on the morphologies and the composition of the film, together with creating a cohesive image of the deposition process for multi-element ceramic coatings. Based on further mechanical and corrosion resistance properties, ceramic film structures are proposed for different industrial applications.

## 4. Conclusions

Pulsed laser deposition of two different ceramics (Al_2_O_3_ + SiO_2_ and Al_2_O_3_ + TiO_2_ + WC) was investigated in order to understand the fundamental mechanisms involved aimed towards scaling the technological process. ICCD fast imaging and optical emission spectroscopy were employed to investigate the kinetics of the laser-produced plasmas. ICCD imaging revealed a splitting of the laser-produced plasmas into three structures, each characterizing a specific species from the plasma (cluster and nanoparticle, atoms and ions). Each structure expands with a different velocity, which is also dependent on the structure and properties of the target. Optical emission spectroscopy revealed that in the laser fluence conditions, the ablated cloud has the same structure as the target, thus leading to quasi-stoichiometric transfer from target to thin film. The plasma generated on a Al_2_O_3_ + TiO_2_ + WC mixture has a larger spatial expansion and was defined by lower kinetic energy when compared with the plasma generated on a Al_2_O_3_ + SiO_2_ mixture. A higher density of emission lines is seen for the plasma containing a wider distribution of elements, attributed to the scattering and collisional processes occurring during pulsed laser deposition. Both individual kinetics and electron excitation temperature were determined for each species. The results provided a clear relation between the atomic mass of the species and their kinetic energy, while the losses through collision and scattering processes were well captured by electron temperature. Investigations into the re-deposited material confirmed a wider angular expansion of lighter elements such as C, O or Al, while heavier elements are defined by a small angle centered on the main expansion axis of the plasma. The ceramic layers obtained through PLD are structurally homogeneous with rare material droplets. A similar proportion of Al and Si were found in the thin films to that obtained on the target, resulting from the influence of the substrate and the percentage variation in oxygen.

## Data Availability

Not applicable.
